# Dedifferentiated endometrial carcinoma metastasis to axillary lymph node: a case report

**DOI:** 10.1186/s13256-023-04192-6

**Published:** 2023-10-30

**Authors:** Chase William Morrison, Kayvon Nick Sanjasaz, Saul David Nathanson, Supriya Raina-Hukku, David Matthew Pinkney, Alexis Anna Davenport

**Affiliations:** 1 Wayne State University, Detroit, USA; 2 Department of Surgery, Henry Ford Health and Wayne State University Medical School, 2799 W Grand Boulevard, Detroit, MI 48202 USA; 3 Department of Pathology, Henry Ford Health, Detroit, MI USA; 4 Department of Radiology, Henry Ford Health, Detroit, MI USA

**Keywords:** Case report, Endometrial carcinoma, Immunotherapy, Metastatic carcinoma, Axillary mass

## Abstract

**Background:**

We present an unusual case of a left axillary lymph node metastasis from a primary dedifferentiated endometrial carcinoma. This pattern of metastasis is likely the result of circulating tumor cells reaching the node through its arterial blood supply.

**Case presentation:**

In this report, a 68-year-old white woman with a dedifferentiated endometrial carcinoma underwent a hysterectomy. She later developed an enlarged axillary lymph node due to metastatic dedifferentiated endometrial carcinoma, treated with chemotherapy and anti-programmed cell death protein 1 immunotherapy resulting in a complete clinical and radiological response.

**Conclusion:**

A review of the literature reveals the rarity of blood-borne lymph node metastasis, especially with uterine carcinoma. Immunotherapy has shown promising results in the treatment of some subtypes of metastatic uterine carcinoma.

## Background

Metastasis of endometrial carcinoma to lymph nodes (LNs) occurs in an anatomically appropriate (pelvic and paraaortic) fashion in half of the cases reported [1], often associated with lymphovascular space invasion. Extra-anatomic LN metastases (mets) in the left supraclavicular fossa, described by Virchow [2], have been reported in patients with carcinoma of the uterine cervix [3] and other intraabdominal primary carcinoma sites [4]. The pathways that uterine cancer cells use to get to these nodes is through lymphatic trunks in the pelvis draining to the appropriate anatomic LNs and the thoracic duct to Virchow’s node in the neck.

Systemic mets to distant LNs, requiring tumor cell transport through the blood circulation, are rarely seen. Isolated reports of axillary LN metastases from gastric [5], lung [6], or endometrial cancers [7, 8] do exist but the pathological and clinical variables responsible for this unusual pattern of spread are unknown. In a large database of the most common locations for mets for stage IV endometrial carcinoma, distant LN metastasis is not mentioned [9].

Endometrial carcinoma has been grouped into four molecular subclasses on the basis of mutational burden, copy number alterations, and driver mutations [10]. The polymerase epsilon-mutated and microsatellite unstable subgroups may represent up to 40% of endometrial cancers, and have been shown to be immunogenic [10].

Endometrial carcinoma is also described on the basis of histology, with the most common type being endometrioid, followed by the more uncommon clear cell and serous cell (both non-endometrioid) [11]. Even more rare is the recently recognized dedifferentiated endometrial carcinoma, which tends to be more aggressive, diagnosed at a later stage, and noted to be resistant to conventional chemotherapies [12]. Several studies have reported deficient mismatch repair (dMMR) or high microsatellite instability (MSI-H) in 46–73% of dedifferentiated endometrial carcinomas [13]. Of note, these dMMR carcinomas have been shown to express programmed cell death protein 1 (PD 1)/programmed cell death ligand 1 (PD-L1) at high rates [14]. This supports the use of immunotherapy in this subset of patients, with recent changes in the systemic management of stage IV endometrial carcinoma having begun to incorporate immunotherapy with promising results [10].

We report a case of dedifferentiated endometrial carcinoma that was initially treated with total abdominal hysterectomy and bilateral salpingo-oophorectomy in the month of October. Paravaginal recurrence was noted on routine follow-up pelvic magnetic resonance imaging (MRI) 4 months later, in February. This patient presented to a breast clinic roughly 40 days later with an axillary lymph node suspect of primary breast cancer. Metastatic workup revealed it to be an unusual pattern of metastatic spread of recurrent endometrial carcinoma, along with local paravaginal recurrence. It was then successfully treated with systemic chemotherapy and PD-1 blockade and the patient’s disease is currently undetectable.

To the best of our knowledge, there exists only eight cases reported in literature of endometrial carcinoma distant metastasis to the breast, which were of clear cell and serous cell histology and not dedifferentiated [13]. This case report presents a unique presentation of a rare subtype of endometrial carcinoma metastasizing to the axillary lymph node with a remarkable response to immunotherapy.

## Case presentation

**Fig. 1:**
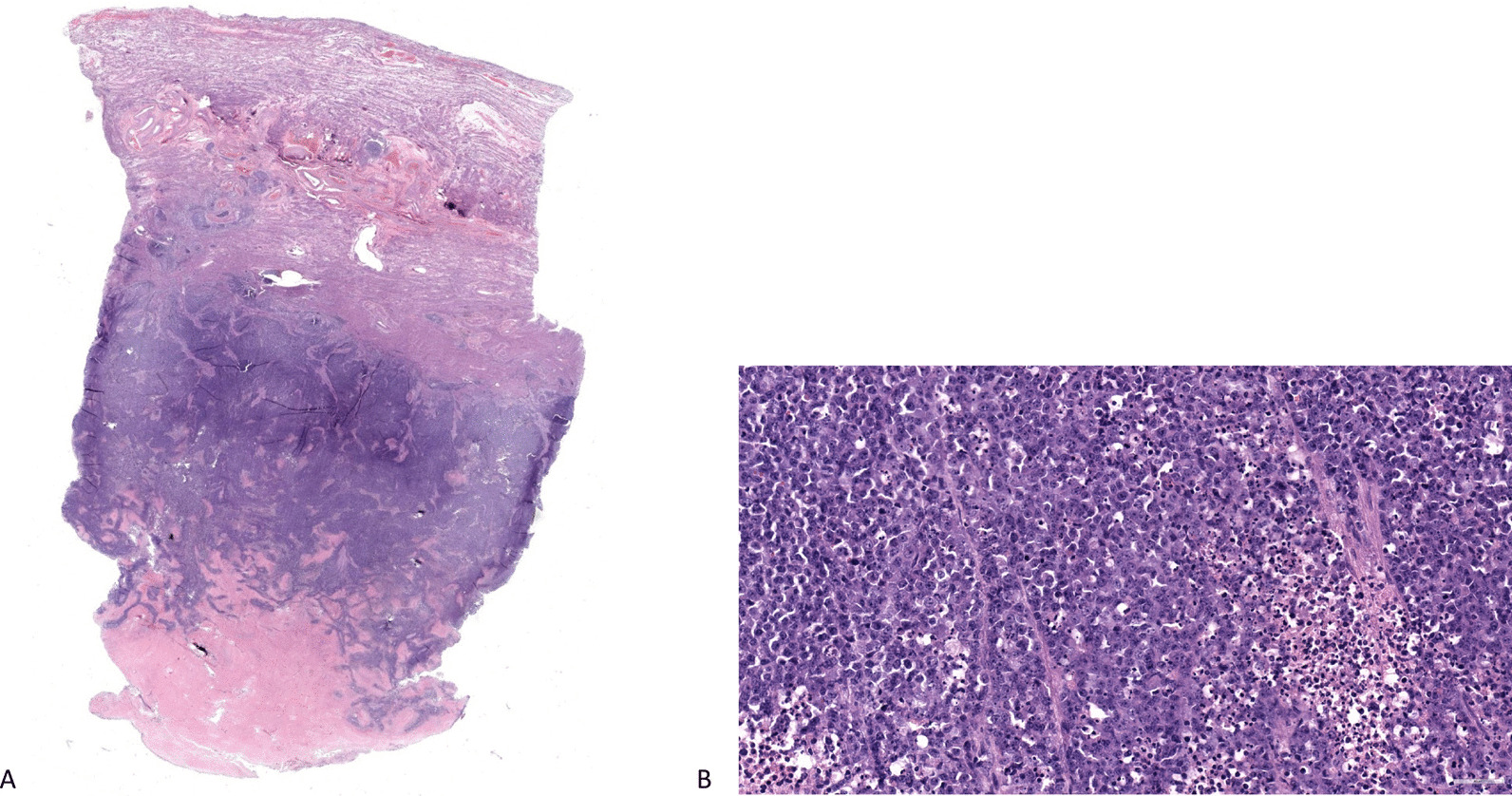
**A** Uterus, full thickness section, 2 ×, hematoxylin and eosin (H&E) stain; tumor extensively infiltrating myometrium (outer half) with lymphovascular invasion. **B** Uterus, 40 ×, H&E stain; tumor composed of diffuse sheets of loosely cohesive neoplastic cells with areas of necrosis; nuclear pleomorphism, prominent nucleoli, and brisk mitoses are seen, consistent with dedifferentiated component of endometrial carcinoma

A 68-year-old white female patient had initially presented to her gynecologist at the age of 68 years with postmenopausal menorrhagia. Social history associated with endometrial carcinoma risk include the use of bioidentical hormone therapy for > 10 years, gravida 1 para 0, first menstrual cycle at age 11 years, menopause at 55 years, and lifetime use of oral contraceptives for 7 years. There is also a family history of Lynch syndrome and colon cancer in the patient’s mother. Transvaginal ultrasound demonstrated a 1.2 cm endometrial stripe with a normal-sized uterus and adnexa. Endometrial sampling showed dedifferentiated endometrial carcinoma; immunohistochemical staining was positive for MSH2 and MSH6, and loss of MLH1 and PMS2 expression, suggesting uterine carcinoma deficient in mismatch repair (dMMR). Peripheral blood genetic testing was negative for mutations associated with hereditary cancer. Computed tomography (CT) of the chest, abdomen, and pelvis was ordered and did not show any signs of metastatic disease. The patient then underwent a total abdominal hysterectomy with bilateral salpingo-oophorectomy and bilateral sentinel LN biopsy in October. The right common iliac, right internal common iliac, and left paraaortic sentinel LNs were collected and found on pathology to be negative for metastatic carcinoma (0/3). The uterus and adnexa were normal in size. Pathology of the surgical specimen showed dedifferentiated endometrial carcinoma with > 50% myometrial invasion with extensive lymphovascular space invasion (Fig. 1). Postoperative therapy with radiation and chemotherapy was discussed in detail and recommended, but was ultimately declined by the patient.

**Fig. 2:**
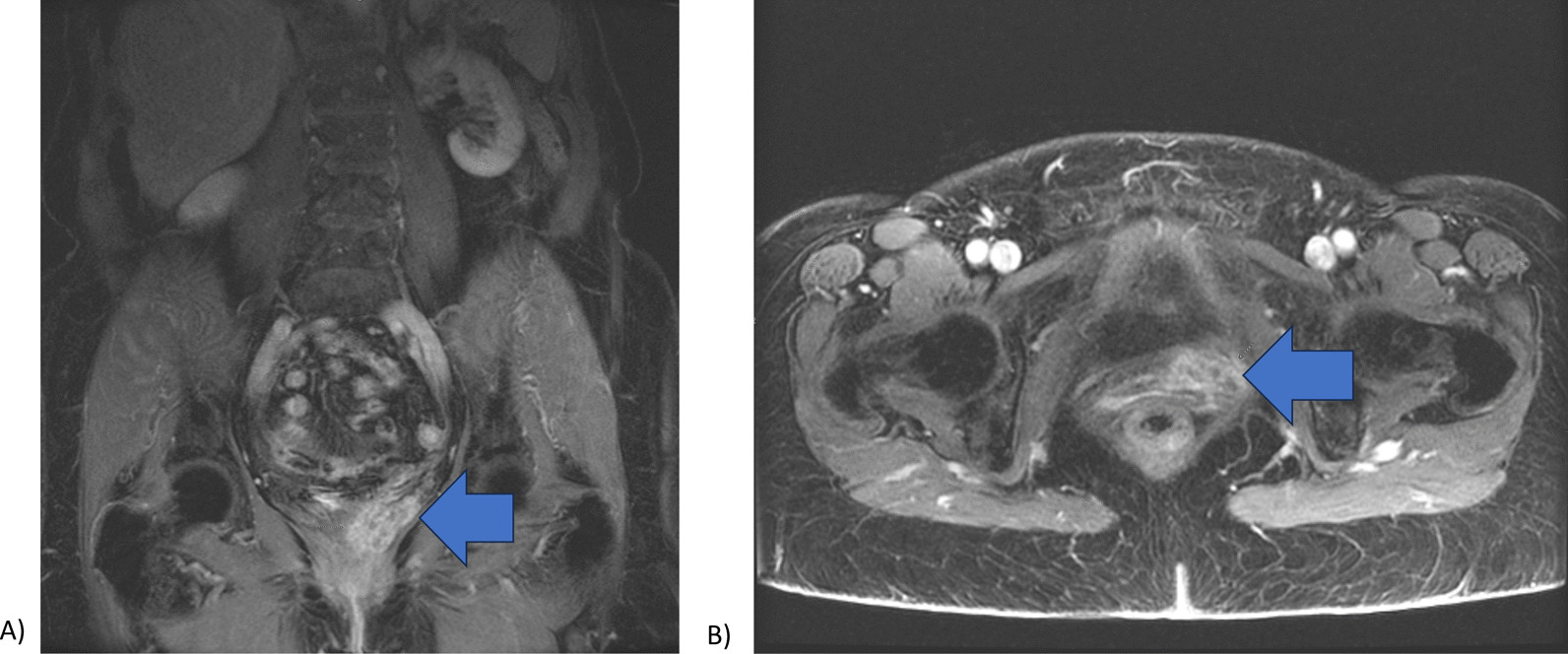
**a** Heterogeneously enhancing structure, suggesting recurrence, found at the anterior vaginal cuff on surveillance CT abdomen and pelvis status post-hysterectomy (arrow) **b** Transverse section of CT pelvis illustrating anterior vaginal cuff recurrence (arrow)

A total of 4 months after hysterectomy, an extra-mucosal mass was palpated in the anterior distal vagina. Surveillance MRI of the abdomen and pelvis illustrated a 2.5 cm heterogeneously enhancing structure at the left anterior vaginal cuff (Fig. 2). Biopsy of the mass showed recurrent dedifferentiated endometrial carcinoma.

**Fig. 3:**
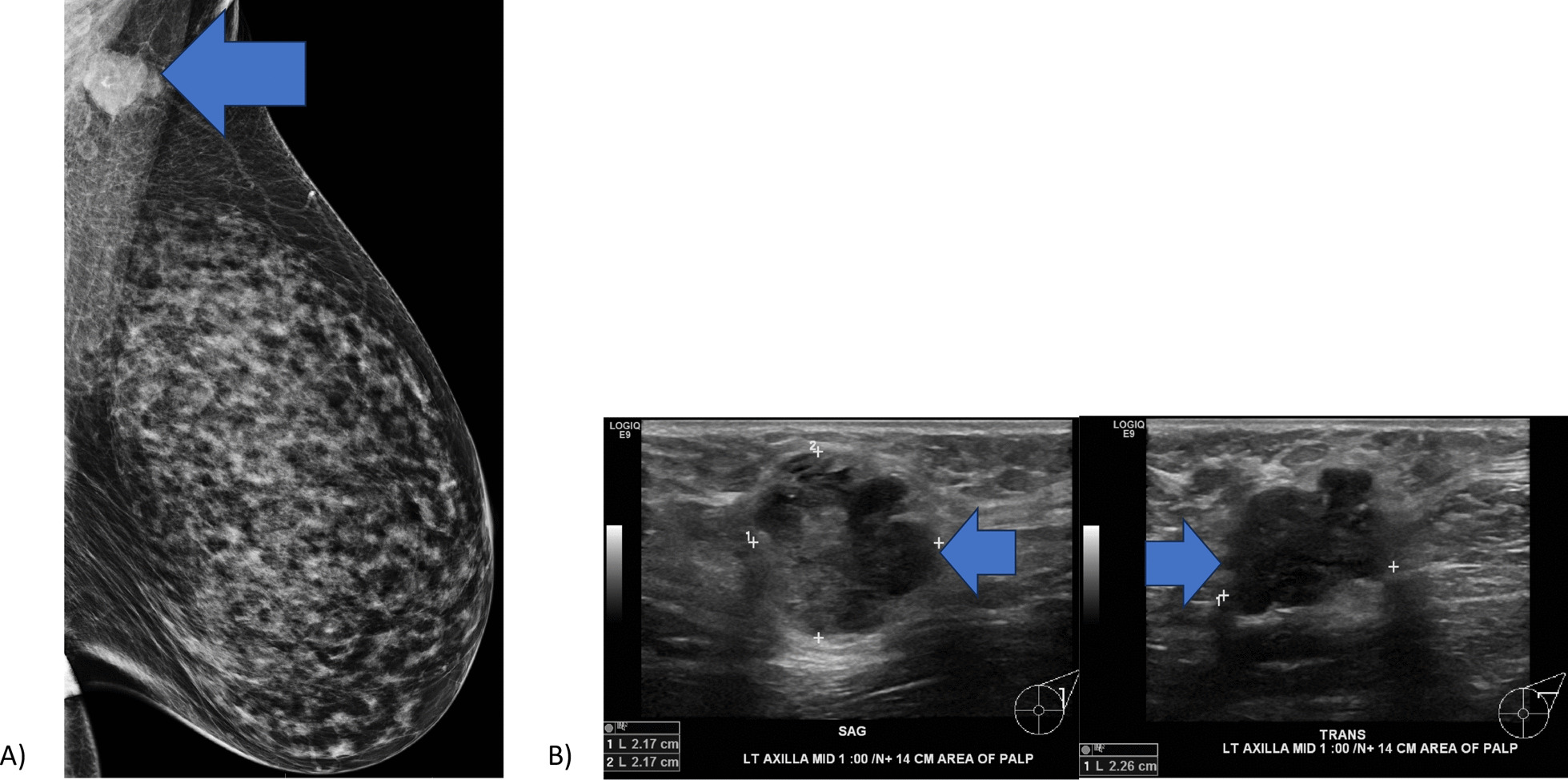
**a** Mammography of the left breast showing axillary LN enhancement (arrow) **b** Targeted ultrasound of the left breast showing hypoechoic regions in sagittal and transverse planes (arrows)

A total of 2 weeks after diagnosis of recurrence, the patient presented to the Breast Center with a tender mass in the left axilla. Physical examination showed normal nipples in both breasts, no palpable breast mass on either side, and no skin abnormalities. A hard, fixed mass, approximately 3 cm in diameter, was palpated high up in the left posterior axilla. Diagnostic mammogram was then ordered, and an irregular mass in the axilla was seen in the mediolateral oblique projection that corresponded to the site of palpable concern (Fig. 3). Targeted ultrasound of the axilla revealed a 2.2 × 2.2 × 2.3 cm mass 14 cm from the left nipple (Fig. 3). The mass exhibited a hypoechoic pattern, angular margins, and irregular shape with vascularity.

**Fig. 4:**
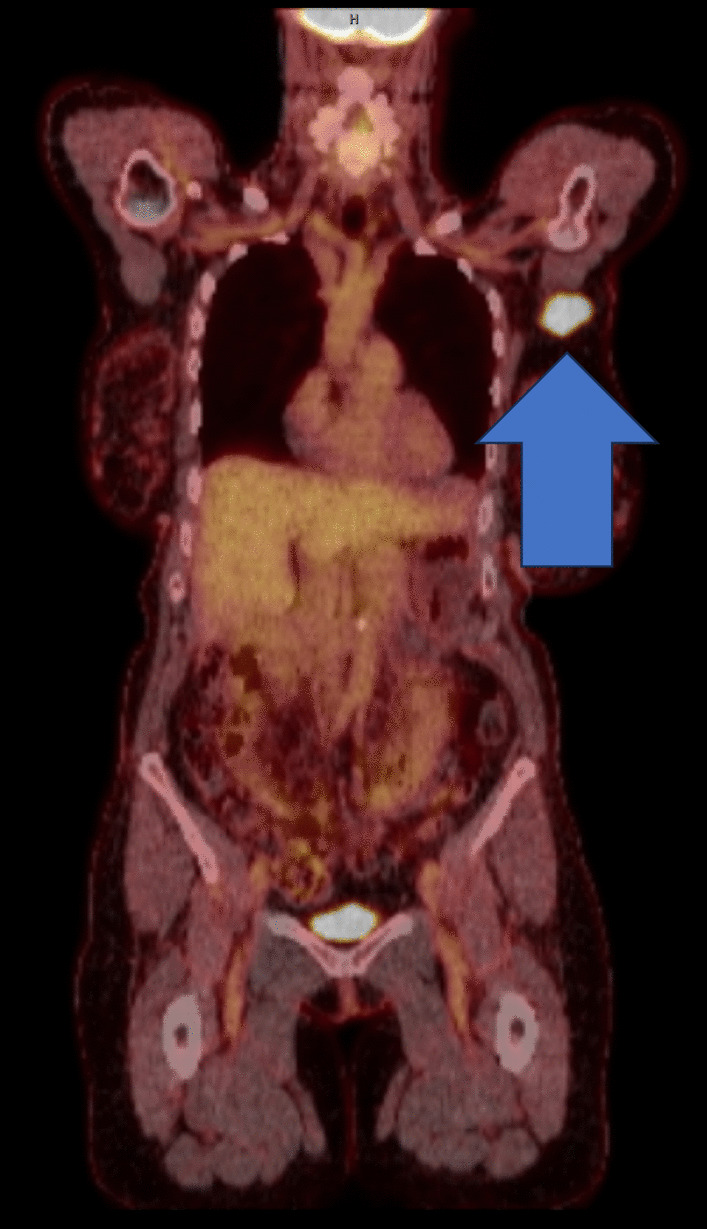
PET-CT showing intense hypermetabolic region in the left axillary LN, arrow illustrating metastatic endometrial carcinoma

A positron emission tomography (PET)-CT scan showed an intensely hypermetabolic 3.3 × 2.3 cm region corresponding to a left axillary LN (Fig. 4). Other hypermetabolic areas included a previously identified paravaginal mass, two pelvic lymph nodes, a 2.6 × 1.1 cm region along the superior medial margin of the left kidney, and a 1.7 × 1.2 cm region in the retroperitoneal fat inferior to the left kidney. Of note, the maximum standardized uptake value (SUVmax) of the axillary mass was 25.7 compared with 3.8 for the perirenal masses. The paravaginal mass was also noted to have a high SUVmax of 25.8.

**Fig. 5:**
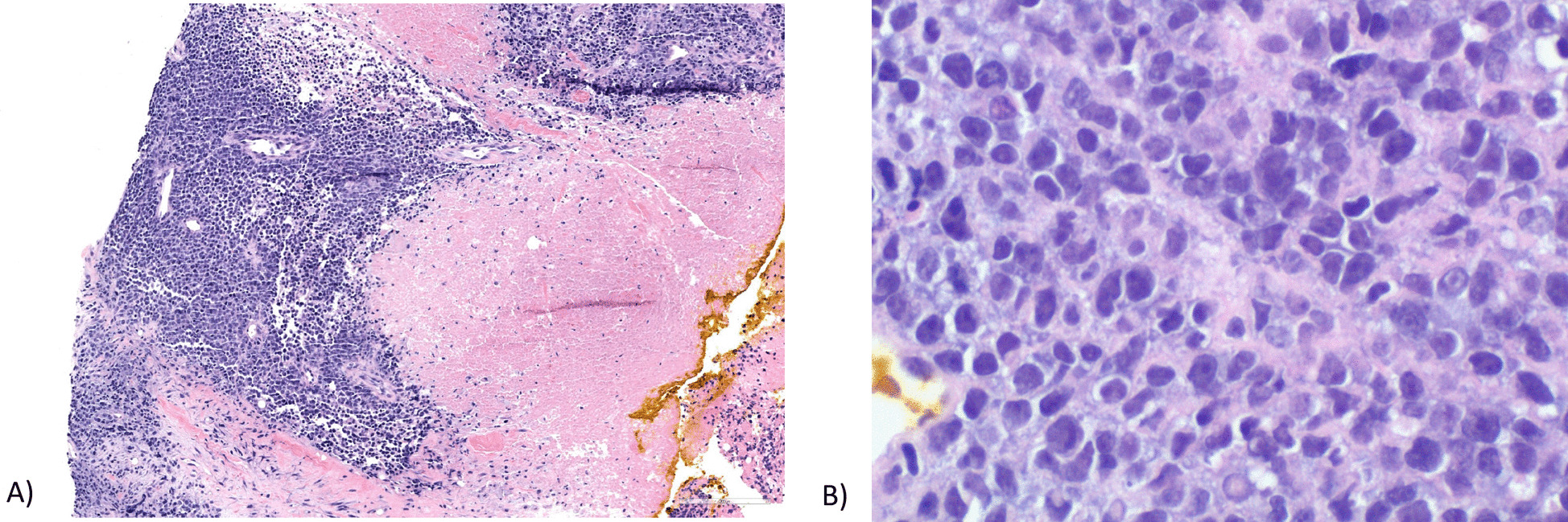
**A** Axillary lymph node 4 ×; H&E stain: needle core biopsy with areas of viable tumor (left) and extensive necrosis (right) **B** Axillary lymph node 40 ×; H&E stain: tumor composed of loosely cohesive neoplastic cells with pleomorphic nuclei and prominent nucleoli brisk mitosis, similar to the dedifferentiated component of primary endometrial carcinoma

A core needle biopsy of the LN was completed and demonstrated a high-grade neoplasm with necrosis (Fig. 5). The morphology of the LN tumor was histologically similar to the dedifferentiated endometrial carcinoma from previous pathology specimens (Fig. 5). Gata binding protein 3 (GATA-3), SRY-Box transcription factor 10 (SOX-10), and estrogen receptor (ER) markers were negative, ruling out primary breast cancer. Metastatic workup was completed within 30 days from her presentation at the breast clinic.

The patient completed four out of six planned cycles of carboplatin (480 mg) and taxol (288 mg) in 3 months. A partial response in the pelvic mass was noted on CT imaging. Chemotherapy was discontinued because of severe neuropathy. Second-line therapy with pembrolizumab 200 mg every 3 weeks was initiated with an undetermined number of total cycles. Five cycles were completed. This patient reported some initial improvement of neuropathy, with some complaints of diarrhea and fatigue.

**Fig. 6:**
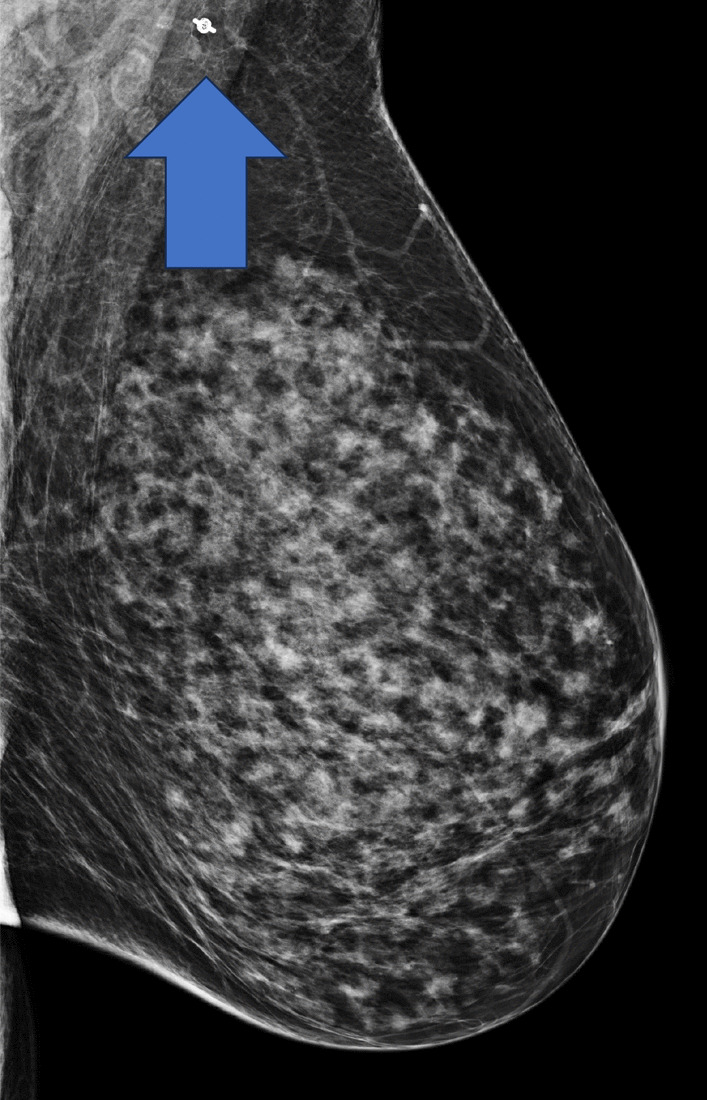
Mammogram status post-pembrolizumab with arrow showing resolution of mass where the metallic biopsy marker is present

Mammography 3 months after pembrolizumab completion showed no evidence of axillary LN disease (Fig. 6). CT also found no evidence of pelvic disease, suggesting a complete radiologic response to the systemic treatment with chemotherapy/immunotherapy. She is currently on routine surveillance.

## Discussion and conclusion

LN mets do occur with endometrial carcinoma. Metastatic tumor cells are usually carried to the LNs from the primary site via lymphatic trunks [13]. The pathway from a primary uterine carcinoma to anatomically appropriate LNs in the pelvis and related locoregional intraabdominal and inguinal LNs is initiated through invasion of peri- or intra-tumoral lymphatic capillaries and flow via lymphatic trunks, from where they enter the sentinel LN subcapsular sinus via afferent lymphatics penetrating the capsule of the LN.

Unusual extra-abdominal sites of LN mets from primary endometrial carcinomas have been reported rarely (0.4–1%) in the supraclavicular or mediastinal LNs [4]. Supraclavicular LN mets are carried to that site via the thoracic duct as described by Virchow for subdiaphragmatic cancers [2] and confirmed more recently by others [15].

A rare form of left axillary LN mets is not via direct lymphatic spread, but rather circulating endometrial tumor cells entering blood vessels either at the site of the primary uterine tumor or by invading high endothelial venules in a LN, gaining access to the systemic circulation [16, 17]. Circulating tumor cells can produce organ metastases, such as the lungs, liver, brain, or bone, but very rarely in distant LNs [18].

Tumor cells circulating in the blood stream have access to any organ in the body, but despite high volume blood supply to organs such as the spleen and skeletal muscle, with associated high concentration of tumor cells, these organs rarely have metastatic deposits. Paget noticed patterns of systemic metastasis in women with breast cancer and thought the reasons why some organs, such as lung, liver, bone, and brain, were more likely to promote metastatic growth were because they provide a ‘soil’ where tumor cells (‘seeds’) could grow [19]. Distant LNs, such as the axilla in relation to the uterus, do not usually provide the appropriate ‘soil’ for mets. Circulating uterine tumor cells must enter the parenchyma via the arterial inflow in the LN hilum. Any of the more common malignancies, with tumor cells circulating in the bloodstream [20], can theoretically metastasize to non-regional LNs such as in the axilla [21], but the literature has very few examples of this phenomenon.

In total, 97% of axillary LN mets originate from the breast [22]. Other locoregional sites of origin include trunk or upper limb skin lesions, such as melanoma or other skin malignancies, including squamous or Merkel cell carcinomas [23]. Axillary LN mets from distant sites include those from gastrointestinal, lung, and ovarian primary tumors [5, 6]. A few cases of endometrial and cervical cancer axillary LN mets have been reported [7, 8].

High-grade metastatic endometrial cancers, especially those with estrogen and/or progesterone receptor-negative disease, are known to have a poor prognosis, rarely producing long-term survival or complete pathologic responses [24]. The cornerstone of treatment for advanced, relapsed, or metastatic disease is chemotherapy with platinum compounds, anthracyclines, and taxanes, often using combination regimens [25]. Targeting of specific molecular pathways, such as mediators of signal transduction in cell proliferation by novel and selective antineoplastic agents in endometrial cancer, is still in its infancy [26].

One subgroup of four molecular subclasses of uterine cancers defined by their driver mutations, mutational burden, and copy number alterations, is highly immunogenic [10]. Polymerase epsilon-mutated and microsatellite-unstable may represent up to 40% of endometrial cancers [10]. Because of the inherent immunogenicity of these MSI-high tumors, combined immune modulation strategies, including chemotherapy, radiation, and immunotherapy and immune checkpoint inhibitor therapy, are being explored to improve treatment outcomes [10].

The 5-year survival rates for patients with endometrial carcinoma involving LNs is < 50%, decreasing to < 20% for those with peritoneal or distant metastasis [27]. Recent studies have found anti-PD1 such as pembrolizumab to be effective in the setting of treated and recurrent advanced endometrial cancers [28]. Pembrolizumab has shown promise for multiple solid tumors and was approved by the FDA in 2022 for treatment of microsatellite instability-high (MSI-H) and mismatch repair deficient endometrial carcinoma that shows progression refractory to prior systemic therapy [29]. Pembrolizumab has shown objective response rates (ORR) of 46%, with a complete response rate of 12% and partial response rate of 33% at a median follow-up of 16 months [30].

Ongoing trials focus on evaluating the possibility of immunotherapy becoming first-line therapy in advanced endometrial carcinoma, exemplifying increased progression-free and overall survival regardless of MMR status [31]. Our patient’s case adds to the current literature a profound response to immunotherapy with pembrolizumab in an atypical metastatic pattern of a rare endometrial carcinoma. Immunotherapy produced a clinical and radiologic complete response that is promising for her long-term outcome. Ongoing studies are important to determine the true value of immunotherapy in the management of endometrial carcinoma spread to distant LNs.

## Data Availability

The data used during the current study are not publicly available due to patient privacy but are available from the corresponding author upon reasonable request.

## Abbreviations

LN: Lymph node
mets: Metastases
